# Increasing hepatic glycogen moderates the diabetic phenotype in insulin-deficient Akita mice

**DOI:** 10.1016/j.jbc.2021.100498

**Published:** 2021-03-02

**Authors:** Iliana López-Soldado, Joan J. Guinovart, Jordi Duran

**Affiliations:** 1 Institute for Research in Biomedicine (IRB Barcelona), The Barcelona Institute of Science and Technology, Barcelona, Spain; 2 Centro de Investigación Biomédica en Red de Diabetes y Enfermedades Metabólicas Asociadas (CIBERDEM), Madrid, Spain; 3 Department of Biochemistry and Molecular Biomedicine, University of Barcelona, Barcelona, Spain

**Keywords:** glycogen, glucose, protein targeting to glycogen (PTG), liver, diabetes, Akita, ATP, glycolysis, gluconeogenesis, insulin, ATGL, adipose triglyceride lipase, FAS, fatty acid synthase, FFA, free fatty acid, GK, glucokinase, GS, glycogen synthase, HFD, high-fat-diet, HSL, hormone-sensitive lipase, PEPCK, phosphoenolpyruvate carboxykinase, PGC1α, proliferator-activated receptor gamma coactivator 1-α, PK, pyruvate kinase, PP1, Protein phosphatase 1, PTG, protein targeting to glycogen, SCD1, stearoyl-CoA desaturase-1, STZ, streptozotocin, TAG, triacylglyceride

## Abstract

Hepatic glycogen metabolism is impaired in diabetes. We previously demonstrated that strategies to increase liver glycogen content in a high-fat-diet mouse model of obesity and insulin resistance led to a reduction in food intake and ameliorated obesity and glucose tolerance. These effects were accompanied by a decrease in insulin levels, but whether this decrease contributed to the phenotype observed in this animal was unclear. Here we sought to evaluate this aspect directly, by examining the long-term effects of increasing liver glycogen in an animal model of insulin-deficient and monogenic diabetes, namely the Akita mouse, which is characterized by reduced insulin production. We crossed Akita mice with animals overexpressing protein targeting to glycogen (PTG) in the liver to generate Akita mice with increased liver glycogen content (Akita-PTG^OE^). Akita-PTG^OE^ animals showed lower glycemia, lower food intake, and decreased water consumption and urine output compared with Akita mice. Furthermore, Akita-PTG^OE^ mice showed a restoration of the hepatic energy state and a normalization of gluconeogenesis and glycolysis back to nondiabetic levels. Moreover, hepatic lipogenesis, which is reduced in Akita mice, was reverted in Akita-PTG^OE^ animals. These results demonstrate that strategies to increase liver glycogen content lead to the long-term reduction of the diabetic phenotype, independently of circulating insulin.

Glycogen is synthesized by glycogen synthase (GS) (1), an enzyme regulated allosterically and by phosphorylation at multiple sites. Protein phosphatase 1 (PP1) catalyzes the dephosphorylation of GS and thus its activation. PP1 is composed of a catalytic subunit (PP1C) and a glycogen-targeting subunit (G subunit). G subunits are molecular scaffolds that localize the catalytic subunit to the glycogen particle (2), where it can interact with enzymes of glycogen metabolism. In mammals, seven G subunits have been identified (PPP1R3A–G), each with a distinct tissue expression pattern (1, 2, 3). Protein targeting to glycogen (PTG) (PPP1R3C or PPP1R5) and G_L_ (PPP1R3B) are expressed in the liver (4), where they promote the storage of glycogen. Accordingly, overexpression of PTG and G_L_ in the liver has been shown to increase glycogen levels (4, 5, 6).

Hepatic glycogen synthesis plays a critical role in maintaining normal glucose homeostasis. After a mixed meal, glucose is taken up by the liver from the portal vein and the systemic circulation and is temporarily stored as glycogen (7). Poorly controlled type 1 diabetic patients exhibit impaired suppression of endogenous glucose production after a meal (8) and a reduction of hepatic glycogen accumulation (9). After short-term (24 h) insulin treatment, glycogen synthesis is improved but not normalized (10). Long-term near normoglycemia, resulting from tight metabolic control, is required to normalize hepatic glycogen metabolism in type 1 diabetic patients. However, the contribution of the indirect (gluconeogenic) pathway of glycogen synthesis remains increased, indicating augmented gluconeogenesis in these patients (11). Impaired GK activity (12, 13) or increased phosphoenolpyruvate carboxykinase (PEPCK) activity (14, 15) could explain this alteration. In rodent models of type 1 diabetes, glycogen stores are depleted, thereby contributing to the development of hyperglycemia (16, 17, 18, 19, 20), and hepatic glycogen is restored to normal levels after insulin treatment (12, 14). In addition to hyperglycemia, disruption of lipid metabolism contributes to the morbidity and mortality associated with patients with diabetes (21). In cases of poor control, patients with insulin-deficient, type 1 diabetes may present with increased plasma triacylglyceride (TAG) levels (22) and ketoacidosis (21). Such hypertriglyceridemia and ketogenesis are stimulated because the increase in adipose tissue lipolysis promotes the delivery of free fatty acids (FFAs) to the liver. Hepatic lipid synthesis is reduced in type 1 diabetic patients (23) and in insulin-dependent diabetic mice (24), and insulin therapy was found to promote lipogenesis (23).

The Akita strain is a mouse model of insulin-deficient and monogenic diabetes. A spontaneous mutation in the insulin 2 gene leads to incorrect folding of the insulin protein, which leads to toxicity in pancreatic ß cells, reduced ß cell mass, and decreased insulin secretion. Heterozygous Akita mice develop insulin-deficient diabetes and show hyperglycemia, hypoinsulinemia, polydipsia, and polyuria by 3 to 4 weeks of age. We previously showed that strategies to increase liver glycogen stores reduce food intake and ameliorate obesity in a high-fat-diet (HFD) mouse model of obesity and insulin resistance (6). This effect was accompanied by a reversal of hyperinsulinemia. It has been reported that pathological hyperinsulinemia drives diet-induced obesity and its complications (25). Therefore, to analyze whether these effects were dependent on the action of insulin, here we used a model with markedly reduced insulin production, the Akita mouse, to examine the long-term effects of increasing liver glycogen on glucose metabolism, food intake, and diabetes complications. We crossed Akita mice with animals overexpressing PTG in the liver (PTG^OE^) (6). The resulting mice (Akita-PTG^OE^) showed increased liver glycogen and long-term reduction of the diabetic phenotype. This observation demonstrates that strategies to increase liver glycogen can provide an effective therapeutic approach to treat insulin-deficient diabetes.

## Results

### Generation of Akita-PTG^OE^ mice

Males heterozygous for the Akita mutation (strain name C57BL/6-Ins2Akita/J) develop hyperglycemia at 3 weeks of age, and this condition becomes progressively more severe with age. The diabetic phenotype is more severe in males than in females (26). Akita mice were crossed with females that overexpressed PTG specifically in the liver (6) to generate the Akita-PTG^OE^ mice. PTG overexpression starts in the second week of embryogenesis and is maintained through the life of the animal. All experiments were performed on males fed *ad libitum*.

### Akita-PTG^OE^ mice showed an improvement of diabetic symptoms

**Figure 1:**
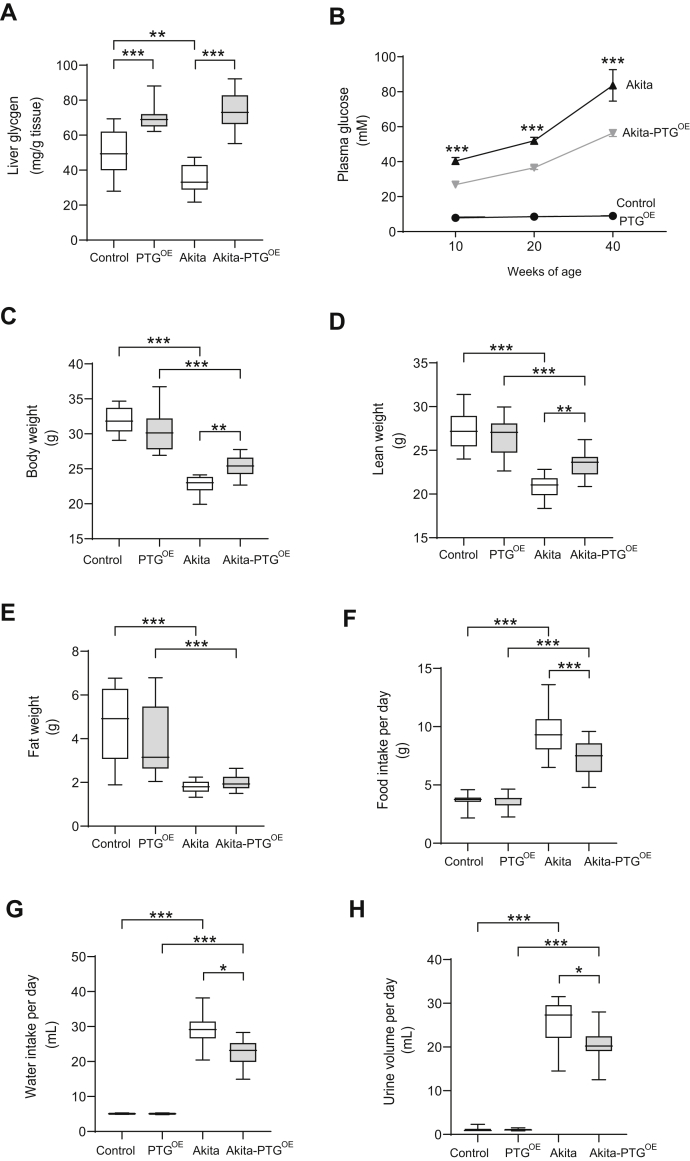
**Fed Akita-PTG**^**OE**^**mice showed an improvement of diabetic symptoms.***A*, liver glycogen, (*B*) plasma glucose concentration at 10, 20, and 40 weeks of age, (*C*) body weight, (*D*) lean weight, (*E*) fat weight, (*F*) food intake per day, (*G*) water intake per day, and (*H*) urine volume per day in Akita, Akita-PTG^OE^, and nondiabetic controls (n = 8–32 in all experiments). Data in *A*, *C*–*H* are from 20-week-old fed animals and are presented in *boxplots*; ∗*p* < 0.05, ∗∗*p* < 0.01, ∗∗∗*p* < 0.001.

**Figure 2:**
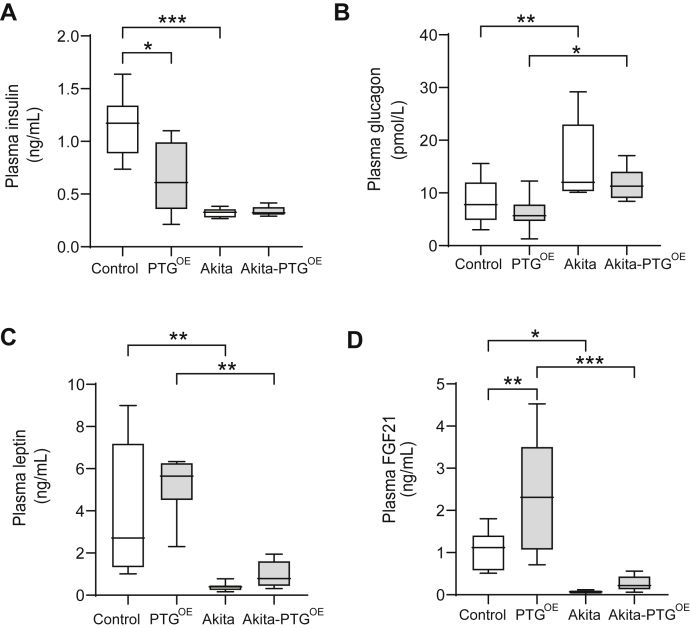
**Plasma hormones were similar in fed Akita and Akita-PTG**^**OE**^**mice.***A*, plasma insulin, (*B*) plasma glucagon, (*C*) plasma leptin, and (*D*) plasma FGF21 in Akita, Akita-PTG^OE^, and nondiabetic controls (n = 7–16 in all experiments). ∗*p* < 0.05, ∗∗*p* < 0.01, ∗∗∗*p* < 0.001.

Liver glycogen is decreased in streptozotocin (STZ)-induced diabetic models (16, 17, 19, 20) and in nonobese diabetic mice (27). Under *ad libitum* feeding conditions, Akita mice showed a 30% reduction in liver glycogen compared with control littermates (Fig. 1*A*). As expected, PTG^OE^ and Akita-PTG^OE^ mice showed a 1.5- to 2.0-fold increase in hepatic glycogen content compared with control and Akita mice, respectively (Fig. 1*A*). Plasma glucose levels were measured in 10-, 20-, and 40-week-old mice fed *ad libitum*. Akita mice showed hyperglycemia with fed plasma glucose levels ∼40 mM at 10 weeks of age, and this hyperglycemia worsened progressively with time. Akita-PTG^OE^ mice showed a 30% reduction in plasma glucose levels compared with Akita mice at the three time points studied (Fig. 1*B*), indicating that hyperglycemia at the onset and during the progression of the disease reduced in response to increased liver glycogen. At 40 weeks of age, the diabetic mice showed extreme hyperglycemia (over 50 mM), decreased activity, and physical deterioration. Therefore, we decided to perform the study at 20 weeks of age. At this age, Akita mice showed a 30% decrease in body weight compared with control mice (Fig. 1*C*), which corresponded to a decrease in lean (25%) and fat mass (60%) (Fig. 1, *D* and *E*). Akita-PTG^OE^ mice were significantly heavier than Akita mice (Fig. 1*C*). This difference in weight was associated with a significantly larger lean mass in Akita-PTG^OE^ mice (Fig. 1*D*). Hyperglycemia in Akita mice was associated with hyperphagia and overt polydipsia and polyuria. Their daily food intake was threefold greater than that of control mice and their fluid consumption was sixfold greater than that of nondiabetic mice. Akita-PTG^OE^ animals ate less (Fig. 1*F*) and showed significant decreases in water consumption (30%) and urine output (25%) compared with Akita mice (Fig. 1, *G* and *H*). We previously demonstrated that strategies to increase liver glycogen content lead to a reduction in food intake and ameliorate obesity and glucose tolerance in a HFD mouse model. These effects were accompanied by a decrease in insulin levels. It was then necessary to check for changes in the circulating levels of insulin and other key hormones in the Akita model. As expected, Akita mice had lower levels of plasma insulin (Fig. 2*A*) and higher plasma glucagon (Fig. 2*B*) than nondiabetic littermates, but these levels were not altered in the Akita-PTG^OE^. Leptin is an adipocyte-derived hormone involved in the regulation of food intake and energy expenditure (28). In insulin-deficient diabetic animals, adiposity and plasma leptin levels are decreased (29, 30). Akita and Akita-PTG^OE^ mice showed a similar reduction in adiposity and plasma leptin levels (Figs. 1*E* and 2C). Fibroblast growth factor 21 (FGF21), which is a potent regulator of metabolism and circulates in the blood (31), was increased in PTG^OE^ mice compared with control mice, and this increase may be attributable to decreased insulin since it has been described that the level of insulin under normal conditions inhibits the production of FGF21 (32, 33, 34). However, the physiological pattern of FGF21 secretion present in healthy individuals is lost in type 1 diabetic patients (33), and this could explain why FGF21 levels are not affected by PTG overexpression in Akita mice (Fig. 2*D*). In summary, the lowering of blood glucose in Akita-PTG^OE^ mice was independent of changes in the levels of circulating insulin, glucagon, leptin, and FGF21.

### Akita-PTG^OE^ mice showed normalized gluconeogenesis and glycolysis in the liver

**Figure 3:**
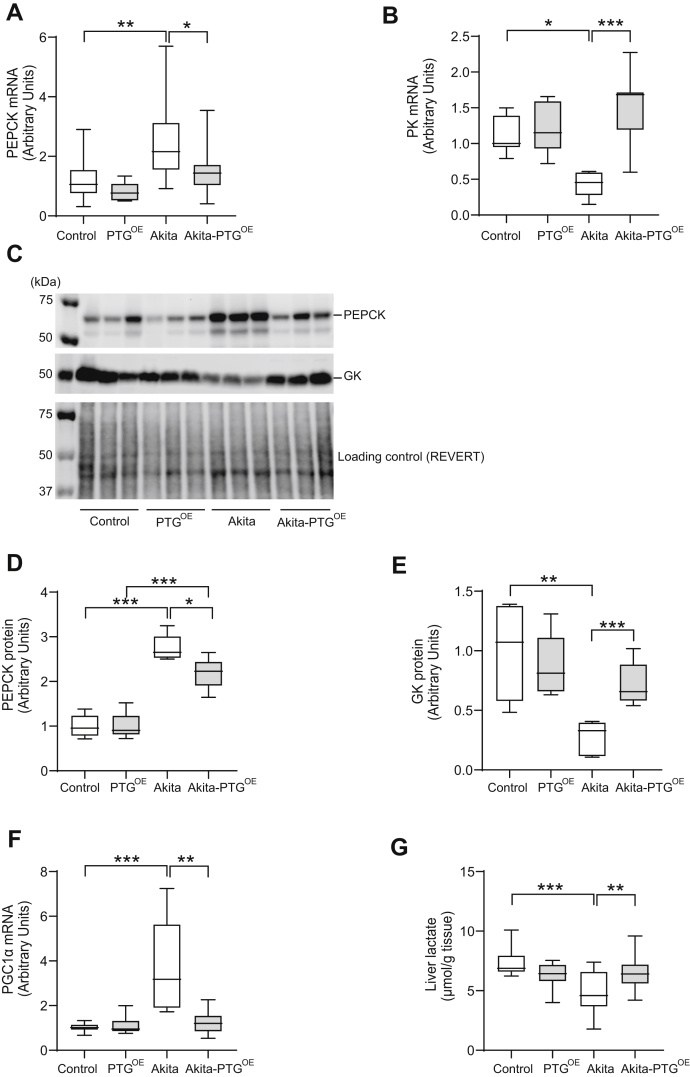
**Akita-PTG**^**OE**^**mice showed normalized gluconeogenesis and glycolysis in the liver.***A*, PEPCK mRNA expression, (*B*) PK mRNA expression, (*C*) representative western blot images of PEPCK, GK, and loading control, (*D*) PEPCK protein expression, (*E*) GK protein expression, (*F*) PGC-1α mRNA expression, and (*G*) hepatic lactate levels in Akita, Akita-PTG^OE^, and nondiabetic controls (n = 6–19 in all experiments). ∗*p* < 0.05, ∗∗*p* < 0.01, ∗∗∗*p* < 0.001.

To determine whether relief of hyperglycemia in Akita-PTG^OE^ mice was associated with changes in hepatic glucose metabolism, we measured the expression of the key enzymes PEPCK, glucokinase (GK), and pyruvate kinase (PK). Akita mice showed an increase in PEPCK and a decrease in GK and PK, thereby indicating an increase in gluconeogenesis and a decrease in glycolysis. Interestingly, Akita-PTG^OE^ mice showed a reversal of these effects (Fig. 3, *A*–*E*). Peroxisome proliferator-activated receptor gamma coactivator 1-α (PGC1α) is a coactivator of several transcriptional factors that regulate the transcription of rate-limiting gluconeogenic enzymes such as PEPCK. PGC1α expression was elevated in the livers of Akita mice but normalized in those of Akita-PTG^OE^ animals (Fig. 3*F*). These changes were associated with an increase in the hepatic content of lactate in the latter model (Fig. 3*G*).

### Akita-PTG^OE^ mice showed restored hepatic lipid metabolism

**Figure 4:**
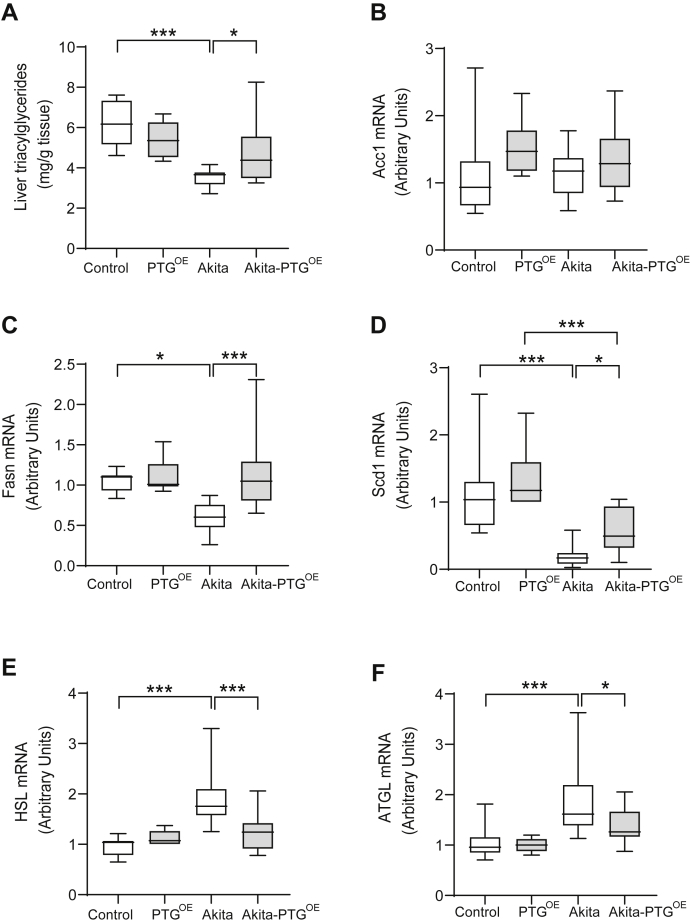
**Akita-PTG**^**OE**^**mice showed restored hepatic lipid metabolism.***A*, hepatic TAG, (*B*) ACC1 mRNA expression, (*C*) FAS mRNA expression, (*D*) SCD1 mRNA expression, (*E*) HSL mRNA expression, (*F*) ATGL mRNA expression in Akita, Akita-PTG^OE^, and nondiabetic controls (n = 6–17 in all experiments). ∗*p* < 0.05, ∗∗*p* < 0.01, ∗∗∗*p* < 0.001.

We next studied the impact of PTG overexpression on hepatic lipid metabolism. Lipogenesis is downregulated in insulin-deficient diabetes. Accordingly, hepatic TAGs were decreased in Akita compared with nondiabetic mice. However, Akita-PTG^OE^ mice showed similar levels of hepatic TAG as nondiabetic animals (Fig. 4*A*). The expression of some of the main lipogenic genes, *i.e.*, fatty acid synthase (FAS) and stearoyl-CoA desaturase-1 (SCD1), was lower in the livers of Akita mice compared with controls (Fig. 4, *C* and *D*), but was upregulated in Akita-PTG^OE^ compared with Akita mice (Fig. 4*D*). Of note, acetyl-CoA carboxylase 1 (ACC1) expression was similar across the groups (Fig. 4*B*). Although normally present at very low levels in the liver (35), hormone-sensitive lipase (HSL) and adipose triglyceride lipase (ATGL) contribute to hepatic TAG hydrolase activity and play a direct role in liver lipolysis (35). Their transcripts are upregulated in the livers of insulin-deficient STZ-diabetic mice (20), and we found the same pattern in the livers of Akita mice (Fig. 4, *E* and *F*). However, Akita-PTG^OE^ animals showed similar levels of HSL and ATGL compared with those of nondiabetic controls (Fig. 4, *E* and *F*).

### Akita-PTG^OE^ mice maintained the hepatic energy state

**Figure 5:**
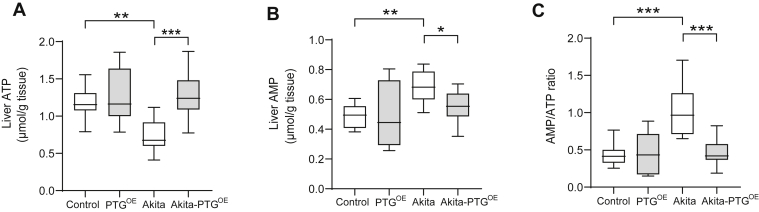
**Akita-PTG**^**OE**^**mice maintained the hepatic energy state.***A*, hepatic ATP, (*B*) hepatic AMP, and (*C*) AMP/ATP ratio (n = 6–20 in all experiments). ∗*p* < 0.05, ∗∗*p* < 0.01, ∗∗∗*p* < 0.001.

Metabolic disorders such as diabetes lead to decreased ATP (36) and also increased AMP levels in the liver (37). Accordingly, the livers of Akita mice had a lower ATP (Fig. 5*A*) and higher AMP (Fig. 5*B*) content compared with control mice, which resulted in a higher AMP/ATP ratio (Fig. 5*C*). In contrast, Akita-PTG^OE^ mice showed levels of adenine nucleotides similar to those of control animals, thus indicating that the hepatic energy state in this diabetic model was improved by increasing liver glycogen.

### PTG overexpression did not modify plasma lipid parameters

**Figure 6:**
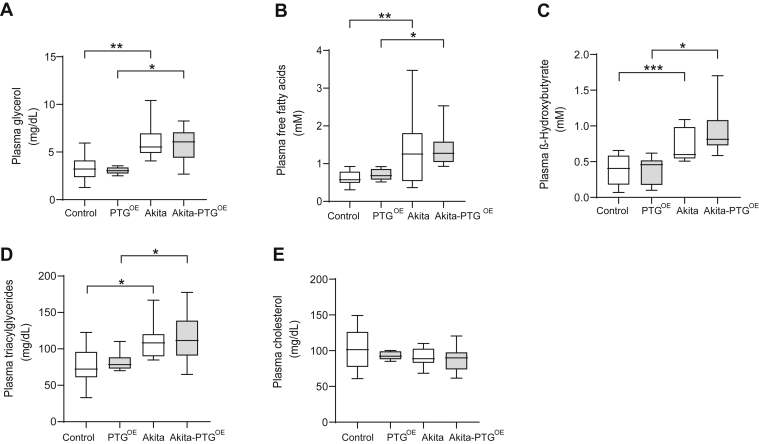
**Plasma metabolites related to lipid metabolism were similar in fed Akita and Akita-PTG**^**OE**^**mice.***A*, plasma glycerol, (*B*) plasma FFA, (*C*) plasma ß-Hydroxybutyrate, (*D*) plasma TAG, and (*E*) plasma cholesterol in Akita, Akita-PTG^OE^, and nondiabetic controls (n = 8–20 in all experiments). ∗*p* < 0.05, ∗∗*p* < 0.01, ∗∗∗*p* < 0.001.

We also measured other plasma metabolites that are altered in diabetes. As expected, plasma glycerol, FFA, β-hydroxybutyrate, and TAGs were increased in diabetic animals compared with their nondiabetic littermates. However, these changes were not corrected in Akita-PTG^OE^ mice (Fig. 6, *A*–*D*). The levels of plasma cholesterol (Fig. 6*E*) were similar between the different genotypes.

## Discussion

The aim of the current study was to evaluate the long-term effects of increasing liver glycogen in the context of insulin-deficient diabetes. We demonstrate that long-term enhancement of liver glycogen reduces hyperglycemia, since Akita-PTG^OE^ mice showed a consistent decrease in plasma glucose that was maintained as the disease progressed. The reduction in hyperglycemia was accompanied by an amelioration of the diabetic symptoms polyphagia, polydipsia, and polyuria. This effect was independent of changes in circulating insulin, glucagon, leptin, or FGF21. Given these findings, an explanation for the observed phenotype must be sought in the changes induced in the liver by PTG overexpression. Akita-PTG^OE^ mice have increased glycogenic capacity. Therefore, the diversion of blood glucose toward the synthesis of glycogen may explain the reduced hyperglycemia. However, another mechanism that may be responsible for the reduction in glucose is the lower gluconeogenic and higher glycolytic capacity observed in the liver of Akita-PTG^OE^ mice. PGC1α has been identified as a key regulator of hepatic gluconeogenesis and its expression is increased in diabetes (38, 39). In Akita-PTG^OE^ mice, we found that PGC1α and PEPCK expression was restored to normal levels. Also, these animals showed normalized GK protein expression, which may also contribute to reducing blood glucose levels. GK plays a central role in the maintenance of glucose homeostasis, and it has been proposed that strategies to increase the hepatic expression or activity of GK in diabetic patients may ameliorate hyperglycemia. In this regard, GK overexpression has been demonstrated to reduce blood glucose levels (40). Akita-PTG^OE^ mice also showed an upregulation of PK gene expression and an increase in hepatic lactate content, which could be a consequence of increased GK in the livers of these animals, as previously shown in hepatocytes of type 2 diabetes rats overexpressing GK (41).

Another interesting observation is that hepatic lipid metabolism was modulated in Akita-PTG^OE^ mice. In this regard, hepatic TAG concentration increased and the expression of FAS and SCD1 was upregulated. The increase in lipogenesis could be due to the increase in hepatic GK, since it has been reported that in the liver enhanced glycolytic flux as a consequence of GK overexpression leads to increased concentrations of glycerol-3-phosphate and malonyl-CoA, the latter a substrate for the novo lipogenesis (40, 42). Moreover, ATGL and HSL expression was decreased in the livers of Akita-PTG^OE^ mice, thereby indicating a lower lipolytic capacity. Thus, the increase in hepatic TAG observed in Akita-PTG^OE^ mice is related to higher lipogenesis and lower lipolysis in the liver.

The lipolysis in the adipose tissue, which is increased in Akita mice, was not affected by PTG overexpression, as the plasma levels of glycerol, FFA, and ketone bodies, all indicators of adipose tissue lipolysis, were similar in Akita and Akita-PTG^OE^ mice. This observation is consistent with the equal loss in fat weight observed in both groups of diabetic mice. The hypertriglyceridemia found in diabetic animals was not corrected in Akita-PTG^OE^ mice since it is due to a decrease in postprandial TAG clearance and not to an increase in hepatic TAG production and secretion (43).

It has been suggested that a decrease in ATP content is associated with an increase in glucose production in the livers of diabetic animals and humans (36, 44, 45, 46). In this study, Akita-PTG^OE^ mice showed a reduction in glucose production and an increase in liver ATP concentration. This observation emphasizes the importance of liver glycogen in the maintenance of hepatic energy charge, as we have recently described (47). Glycolysis is a major pathway eliciting ATP. The key rate-limiting enzymes for this pathway include GK and PK (48). The increase in the hepatic ATP content in Akita-PTG^OE^ mice could be due to an increase in the expression of GK and PK in the liver promoting the generation of energy. The reduction in gluconeogenesis, an ATP-consuming pathway, may also contribute to the increase in ATP.

Another interesting finding was that Akita-PTG^OE^ mice had a lower food intake compared with Akita mice. Our results indicate that leptin or insulin did not contribute to the hypophagic effect since both hormones were similarly reduced in Akita and Akita-PTG^OE^. The decrease in food intake could be explained by the maintenance of the hepatic energy state observed in Akita-PTG^OE^ mice, since we have demonstrated that the regulation of food intake by liver glycogen (6) is dependent on hepatic ATP signaling to the brain through the hepatic branch of the vagus nerve (49).

In conclusion, we demonstrate that the long-term enhancement of liver glycogen reduces the diabetic phenotype by partially restoring the alterations in hepatic glucose and lipid metabolism induced by insulin-deficient diabetes. These findings expand on previous observations made by our group (16) and others (17) showing that a short-term increase in hepatic glycogen content achieved by adenovirus-mediated overexpression of an activated form of liver GS (16) or of a truncated targeting subunit of PP1 (GMΔC) (17) reduces diabetes symptoms in STZ diabetic rats. However, both studies were performed using adenovirus for short-term hepatic overexpression of the proteins. This experimental approach had two important limitations: first it caused a huge peak in the levels of the exogenous protein, and second, the timeframe of the observation was limited to only 1 week. In the present work, we show that the treatment is effective in the long term. Increasing liver glycogen corrects many of the metabolic disturbances associated with insulin-deficient diabetes, and most importantly, it does so independently of the levels of circulating insulin. On the basis of these observations, the activation of glycogen deposition emerges as a potential therapeutic target for the treatment of insulin-deficient diabetes.

## Experimental procedures

### Mice

All procedures were approved by the Barcelona Science Park’s Animal Experimentation Committee and carried out in accordance with the European Community Council Directive and the National Institute of Health guidelines for the care and use of laboratory animals. Akita mice (strain name C57BL/6-Ins2^Akita^/J) were purchased from Jackson Lab. Heterozygous males were crossed with females that overexpressed PTG specifically in the liver. Mice that overexpressed PTG were generated as previously described (6). Briefly, the PTG cDNA under the control of the ubiquitous CAG promoter (CMV immediate early enhancer/chicken β-actin promoter fusion) was introduced into an innocuous locus by homologous recombination. A loxP-flanked transcription stop cassette was included between the CAG promoter and the PTG cDNA to allow the expression to be dependent upon the action of a Cre recombinase. The resulting mouse line was bred with an albumin promoter Cre recombinase-expressing animal (The Jackson Laboratory), which drove the expression of PTG specifically to the liver.

All experiments were performed in males because they develop a more severe diabetic phenotype. All the mice studied were littermates. Except for Figure 1*B*, animals were sacrificed at 20 weeks of age under *ad libitum*-fed conditions between 8 and 10 AM by cervical dislocation and tissues were collected and frozen in liquid nitrogen. Whole blood was collected from the tails in EDTA-coated tubes and then centrifuged and plasma was stored at −20 °C for analysis.

### Food consumption

To monitor food intake, mice were housed individually and acclimatized for 1 week before study. Food intake was measured daily for five consecutive days.

### Blood and biochemical analysis

Liver glycogen was determined as previously described (47). Hepatic nucleotides (ATP and AMP) were measured by HPLC in perchloric acid extracts, as previously described (49). Liver lactate was measured in perchloric acid extracts using a commercial spectrophotometric kit (Horiba, ABX). Hepatic TAG was quantified in 3 mol/l KOH and 65% ethanol extracts following the method described by Salmon and Flatt (50) and using a kit (Sigma-Aldrich). Plasma insulin, glucagon, leptin (Crystal Chem) and FGF21 (Biovendor, Brno, Czech Republic) were analyzed by ELISA. Plasma glycerol, TAG, and ß-Hydroxybutyrate were measured using a commercial kit (Sigma-Aldrich). Plasma glucose and cholesterol were determined using a commercial kit (Horiba, ABX). FFAs were measured using a commercial kit (Abcam).

### Western blot analysis

Liver samples were homogenized in 50 mM Tris/HCl (pH 7.4), 150 mM NaCl, 1 mM EDTA, 5 mM sodium pyrophosphate, 1 mM sodium orthovanadate, 50 mM NaF, 1% NP-40, 1 mM PMSF, and a protease inhibitor cocktail tablet (Roche). Protein concentration was measured using the bicinchoninic acid (BCA) protein assay reagent (Thermo Fisher Scientific). Immunoblot analysis of homogenates was performed using the following antibodies: PEPCK (a kind gift from Dr E. Beale) dilution 1:100.000 and GK (peptide 414–428 raised by Genosys dilution 1:1000). Proteins were detected by the ECL method (Immobilon Western Chemiluminescent HRP Substrate, Millipore, Sigma-Aldrich). The loading control of the WB membrane was performed using the REVERT total protein stain.

### RNA extraction and quantitative RT-PCR

Liver RNA extraction, RT-PCR, and quantitative real-time PCR analysis were performed as previously described (51). The following TaqMan probes (Applied Biosystems) were used for quantitative real-time PCR: PEPCK (Mm00440636_m1); PK (Mm00443090_m1); PGC1α (Mm00447180_m1); FAS (Mm00662319_m1); SCD1 (Mm00772290_m1); ACC1 (Mm01304257_m1); HSL (Mm00495359_m1); ATGL (Mm00503040_m1); and 18S (Mm03928990_g1). 18S was used as a housekeeping gene.

### Lean body mass and fat mass

Lean body mass and fat mass were measured by magnetic resonance imaging (EchoMRI LLC).

### Statistics

Data are presented in boxplots; on each box, the central mark indicates the median, and the bottom and top edges of the box indicate the 25th and 75th percentiles, respectively. The whiskers extend to the most extreme data points. *p* values were calculated using two-way ANOVA with post hoc Tukey’s test as appropriate.

## Data availability

The data are available on request from the authors.

## Conflict of interest

The authors declare no conflict of interest.
